# Anxiety and Depression in Women Newly Diagnosed with Breast Cancer and Waiting for Surgery: Prevalence and Associations with Socio-Demographic Variables

**DOI:** 10.3390/medicina57050454

**Published:** 2021-05-07

**Authors:** Cristina Civilotti, Rossana Botto, Daniela Acquadro Maran, Brigitta De Leonardis, Beatrice Bianciotto, Maria Rosa Stanizzo

**Affiliations:** 1 Department of Psychology, Università di Torino, 10124 Torino, Italy; cristina.civilotti@unito.it (C.C.); beatrice.bianciotto@edu.unito.it (B.B.); 2 Clinical Psychology Unit, Department of Neuroscience, University of Turin, “Città della Salute e della Scienza” Hospital of Turin, 10126 Turin, Italy; rossana.botto@unito.it (R.B.); brigitta.leonardis@edu.unito.it (B.D.L.); mstanizzo@cittadellasalute.to.it (M.R.S.)

**Keywords:** breast cancer, anxiety, depression, distress, socio-demographic characteristics, diagnosis

## Abstract

*Background and Objectives:* Cancer is a threatening-life disease with a significant psychological burden. The psychological morbidity varies according to the phases of the illness and is influenced by multiple socio-demographic factors, that are useful to consider in order to identify the categories of patients most at risk of developing psychiatric disorders. The present study analyzes, in a sample of women newly diagnosed with breast cancer, the relationships between their levels of anxiety and depression and several socio-demographic characteristics. The study was cross-sectional. *Materials and Methods*: Four hundred and seventy eight women newly diagnosed with breast cancer completed the Hospital Anxiety and Depression Scale during the pre-surgical phase. *Results*: Findings show that almost 40% of the sample had clinically relevant anxious symptoms and about a quarter of the sample had significant depressive symptoms. Their prevalence was higher in widows. Moreover, depressive symptoms were higher in older women and anxious symptoms were higher in patients with a lower educational level. In the pre-surgical phase, women can suffer from clinically relevant anxiety and depression, especially the widows, older women, and women with a lower educational level. *Conclusions*: Identifying the most psychologically vulnerable patients, due to specific socio-demographic characteristics, is essential in order to provide adequate psycho-oncological treatments to the categories of patients, who are most at risk of developing psychopathological concerns.

## 1. Introduction

Every year worldwide, 1.7 million women are diagnosed with breast cancer, that is now recognized as a severe global problem [1]. In Italy, the estimated number per year of new cases of breast cancer is 52,800 [2]. Cancer is a life-threatening disease that can generate a certain grade of disability and entails treatments with a potential strong impact on the individual. According to the literature, more than one-third of breast cancer patients suffer from psychiatric disorders, and anxiety and depression are the most experienced psychological symptoms [3]. Therefore, screening for psychological symptoms and promptly identifying the patients most at psychopathological risk are an essential part of the psycho-oncological interventions. In this regard, the Hospital Anxiety and Depression Scale (HADS; [4]) is a valid tool for detecting anxious and depressive symptomatology in patients with medical conditions and it is considered as one of the best distress-screening instruments currently available for cancer patients [5]. Longitudinal studies evidenced that distress tends to be higher immediately after the diagnosis than in the successive phases [6]: Receiving the diagnosis for the first time represents a meaningful moment with a specific impact on the individual in terms of emotional consequences and disturbance to the life balance. According to Villar et al. [3], the psychological morbidity in cancer patients is influenced by multiple socio-demographic factors that are useful to consider in order to identify the categories most at risk of developing psychiatric disorders. In women with breast cancer, anxiety is higher in the youngest, in those with poor social support, with negative marital relationships and poor communication with partners [7]. Similarly, depressive symptoms are predicted by young age, lack of social support, absence of a stable relationship, and low level of education [8,9]. The present study analyzes, in a sample of women newly diagnosed with breast cancer, the relationships between their levels of anxiety and depression and socio-demographic characteristics, in order to identify those categories of patients that are most at risk for developing psychopathological disorders.

## 2. Materials and Methods

### 2.1. Sample

**Figure 1:**
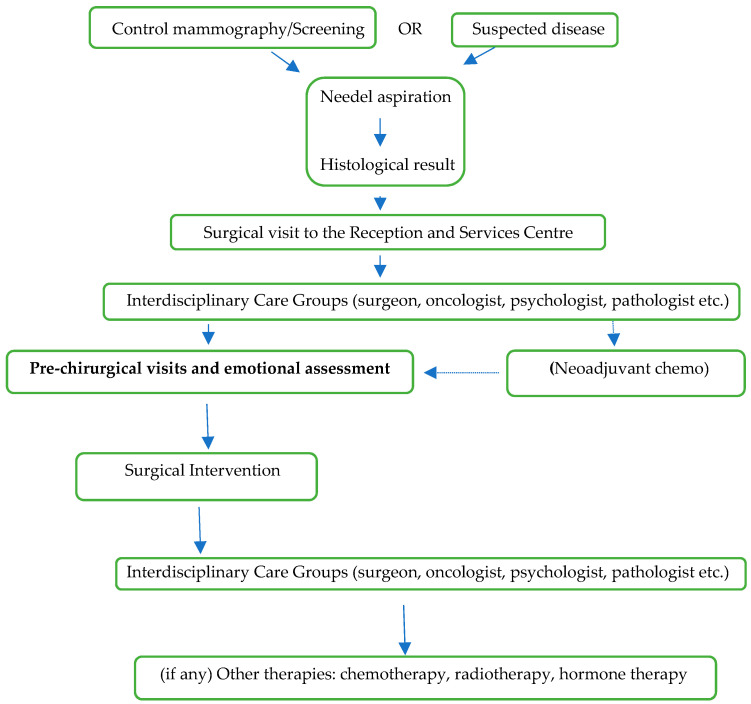
Plan of the cancer diagnosis.

The participant’s eligibility criteria included being female, aged 18 or more, being newly diagnosed of breast cancer and not having previously received a cancer diagnosis, not having yet undergone the breast surgery, as well as not reporting any current psychiatric diagnosis or cognitive deficits, which imply an inability to provide valid informed consent and responses to the study tools. The research was limited to women newly diagnosed and receiving a cancer diagnosis for the first time in order to exclude biases generated by being in cancer recurrence or having had a previous cancer, that could influence the patients’ psychological state. As shown in Figure 1, at the time of administration of the scales, these women had already received the result of the histological examination, following a screening or for a condition. They had already met the surgeon and were waiting for surgery. The evaluation of the emotive state of the patients in this phase of the care is considered a best practice in psycho-oncological intervention [10].

Of the 539 consecutive recruited patients, 61 have been excluded from the study due to incomplete data, refusal or not responding to the criteria. The final sample was composed of 478 women newly diagnosed of breast cancer.

### 2.2. Measures

The Italian version of the Hospital Anxiety and Depression Scale (HADS) has been used for the study [11]. The HADS is a 14-item self-report questionnaire that was originally developed to evaluate the presence of anxiety and depression in the context of a medical non-psychiatric outpatient clinic over the preceding week [4,12] and is nowadays one of the most used tools to evaluate anxiety and depression in cancer patients as a first screening [4,13,14,15]. It is easy with a rapid administration procedure and it has a good reliability [16], as we found in the present study (Cronbach α: 0.898). HADS is divided into two 7-item subscales, HADS-A for anxious symptomatology (an example of the anxiety sub-scale item is: “I feel tense or ‘wound up’”) and HADS-D for depressive symptomatology (an example of the anxiety sub-scale item is: “I still enjoy the things I used to enjoy”). Each item scores on a 4-point Likert scale (e.g., as much as I always do (0); not quite so much (1); definitely not so much (2); and not at all (3), giving maximum subscale scores of 21 for anxiety and depression, respectively. A score of 8 or more in the HADS-A and HADS-D suggests a clinically relevant anxious/depressive symptomatology [17].

### 2.3. Procedure

The study was conducted in accordance with principles embodied in the Declaration of Helsinki. The study was approved by “Comitato Etico Interaziendale A.O.U. San Giovanni Battista di Torino A.O. C.T.O./Maria Adelaide di Torino” (no. 0073054-255/15). The study was performed in accordance with the ethical standards as laid down in the 1964 Declaration of Helsinki and its later amendments. Participants were recruited in the Breast Unit of “Città della Salute e della Scienza” hospital of Turin, during the pre-surgical visits. Informed consent was obtained from all individual participants included in the study. The nurses of the unit gathered the patients’ socio-demographic and clinical data and delivered the HADS to patients. The considered socio-demographic factors were age, marital status, education, occupation, and presence of children.

### 2.4. Data Analysis

Descriptive statistics, i.e., means, standard deviations, and frequencies were performed. Associations between HADS, and age were assessed through Pearson’s correlation index. The *X*^2^ test with a post hoc test was used to evaluate the associations between the presence of clinically relevant anxiety or depression and marital status, education, occupation, and presence of children. About the post hoc test, 1.96 has been considered as the critic value to test the hypothesis. *p* ≤ 0.05 has been considered statistically significant. If significant, a one-way ANOVA was then conducted to determine if HADS-A, HADS-D, and HADS-tot scores were different for socio-demographical variables. All of the statistical analyses were conducted using SPSS version 22.0 (IBM SPSS Statistics for Macintosh, Armonk, NY, USA: IBM Corp.).

## 3. Results

Table 1 presents the socio-demographic characteristics of the sample. In terms of anxious and depressive symptomatology, 39.5% (*n* = 189) of patients were above the cut-off for HADS-A, 26.2% (*n* = 125) of patients were above the cut-off for HADS-D, and 44.2% (*n* = 211) of patients were above the cut-off for HADS-tot.

### Associations of HADS with the Socio-Demographic Variables

**Figure 2:**
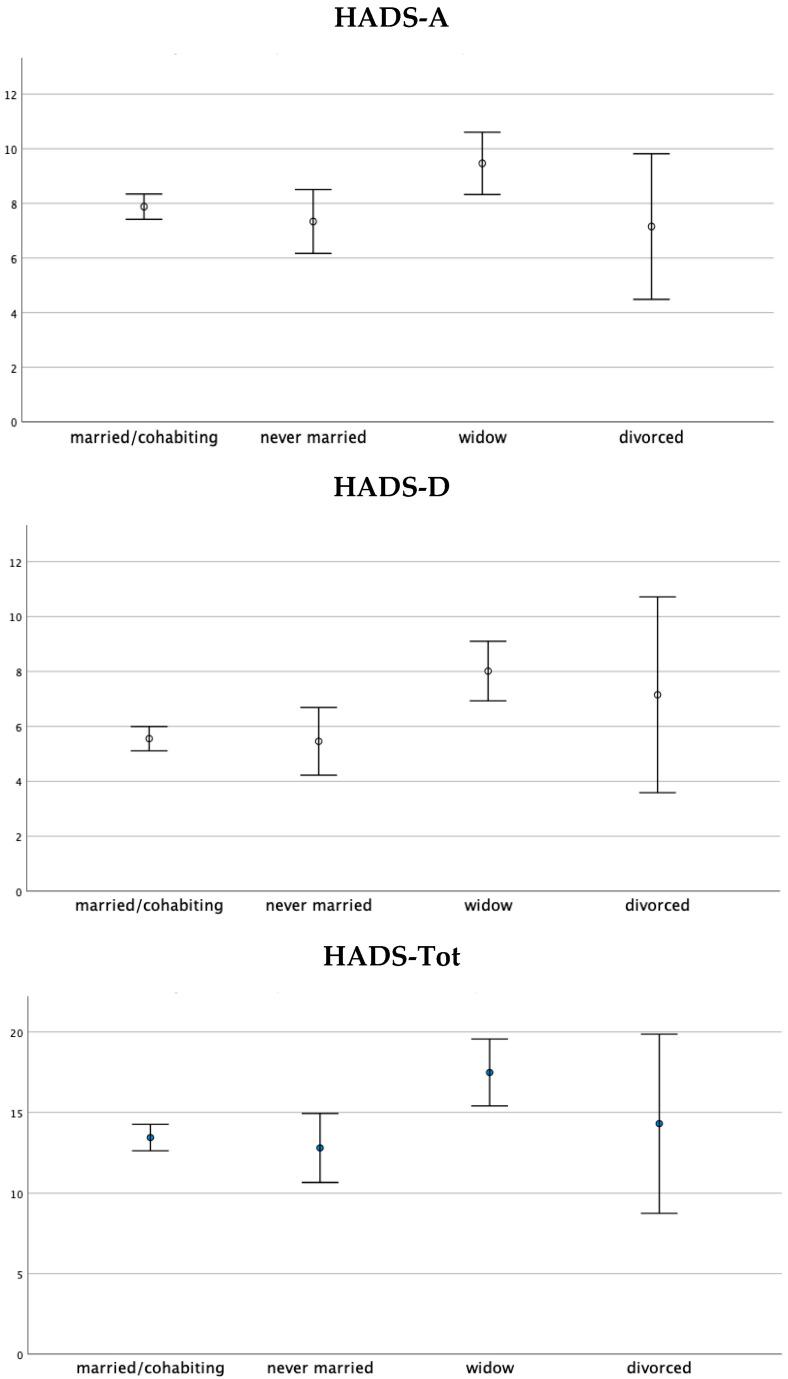
One-way ANOVA on the HADS-A, HADS-D, and HADS-Tot scores with respect to the marital status group. Note: Data are presented as the mean ± standard deviation. The HADS-A, HADS-D, and HADS-Tot scores were significantly different between the marital status groups (respectively, F(3, 419) = 3.258, *p* < 0.05; F(3, 419) = 6.657, *p* < 0.01; and F(3, 419) = 5.381, *p* < 0.01).

Age positively correlated with HADS-D scores (*r* = 0.17, *p* = 0.001). The *X*^2^ test showed a significant association between the marital status and the presence/absence of clinically relevant anxious symptoms (scores ≥ HADS-A cut-off) (*X*^2^ = 13.017, *p* = 0.005) and depressive symptoms (scores ≥ HADS-D cut-off) (*X*^2^ = 8.725, *p* = 0.033). In detail, in the “widow” marital status category, the frequency of patients above the cut-off for HADS-A and HADS-D was higher than those expected in the case of a non-significant association (HADS-A: Adjusted residue: +3.2, 57.35%, *n* = 39; HADS-D: Adjusted residue: +2.4, 38.23%, *n* = 26). Therefore, the frequencies of widowers with clinically relevant anxious and depressive symptoms were higher than the married (HADS-A: 38.25%, *n* = 127; HADS-D: 23.79%, *n* = 79), singles (HADS-A: 31.35%, *n* = 20; HADS-D: 21.87%, *n* = 14), and divorced (HADS-A: 21.42%, *n* = 3; HADS-D: 42.85%, *n* = 6) (Table 2 and Table 3). From the one-way ANOVA, the HADS-A, HADS-D, and HADS-tot scores emerged and were significantly different between the marital status groups (Figure 2).

**Figure 3:**
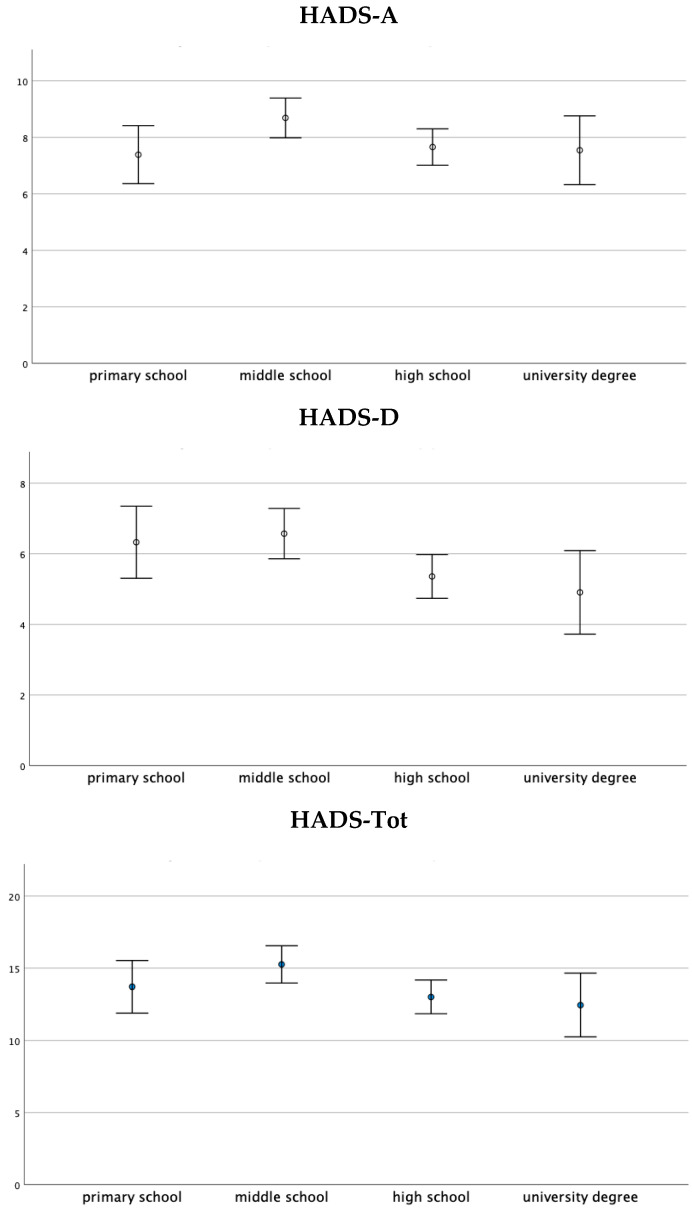
One-way ANOVA on the HADS-A, HADS-D, and HADS-Tot scores with respect to the education level group. Data are presented as the mean ± standard deviation. The HADS-A, HADS-D, and HADS-Tot scores were significantly different between the education level groups (respectively, F(3, 416) = 2.296, *p* < 0.05; F(3, 416) = 3.277, *p* < 0.05; and F(3, 416) = 2.832, *p* < 0.05).

The *X*^2^ test showed a significant association between the level of education and the presence/absence of clinically relevant anxious symptoms (scores ≥ HADS-A cut-off) (*X*^2^ = 11.792, *p* = 0.008). In detail, in the “middle school diploma” level of education category, the frequency of patients above the cut-off for HADS-A was higher than those expected in the case of a non-significant association (HADS-A: Adjusted residue: +2.6, 47.30%, *n* = 167). Therefore, the frequency of patients with a middle school level of education with clinically relevant anxious symptoms was higher than those with a primary school diploma (44.15%, *n* = 34), high school diploma (34.61%, *n* = 63), and with a degree (25%, *n* = 13) (Table 4). From the one-way ANOVA, the HADS-A, HADS-D, and HADS-tot scores emerged and were significantly different between the education level groups (Figure 3).

The occupational status and presence of sons were not significantly associated with the HADS-A and HADS-D scores.

## 4. Discussion

Regarding the results of total HADS, almost 45% of patients reported above the cut-off score for mixed anxiety-depressive symptoms. Looking separately at the two sub-scales of Anxiety and Depression, almost 40% of the sample had clinically relevant anxious symptoms and about a quarter of the sample had significant depressive symptoms. These results are in line with the literature [18]. Regarding anxiety, in the phase of the illness taken into account, i.e., the period between the diagnosis and the treatments, high levels of anxiety are typically connected to the awareness that undergoing surgery is necessary and to the associated emotions, such as fear for the intervention itself or worries for the anesthesia-related aspects [19]. Patients can also be overwhelmed and distressed for many clinical consultations and have to handle a lot of new and complex medical information. Considering which factors are not related to the illness, i.e., socio-demographic characteristics or social aspects, enhancing the probability of psychological distress, allows identifying those categories of patients that, in this initial phase of the illness, are most at risk of developing psychopathological disorders, and better orienting the psychological interventions. In this regard, concerning the marital status, the results of the study evidenced that in widows the presence of anxious and depressive clinically relevant symptomatology was higher than in the other categories. Having a partner from whom receiving support and with whom sharing emotions and thoughts, discussing, deciding, and facing the visits and the treatments can be a resource. It is known that social support is associated with lower levels of depression [20]. Moreover, having a partner at home is a protective factor for the elderly with cancer with respect to the development of psychological symptoms [21]. Effectively, Zhai et al. [22] showed that, differently from widowed patients, the married ones have greater social support, less psychological distress, a better prognosis, less dysfunctions of the immune system, and greater financial resources. Furthermore, at the diagnosis, their risk of dying appeared lower than the unmarried patients [23]. The research revealed that older participants had higher levels of depression. It is possible that in older people cancer occurs in comorbidity with other clinical conditions, thus with a greater impact on the individual’s psycho-physical well-being. In fact, advanced age is recognized as a risk factor for the onset of depression [24] and the cancer diagnosis can be an additional compromising event. Moreover, the development of problems of aging, comorbid symptoms, and the loss of loved ones can make cancer patients more vulnerable to depression. Furthermore, older breast cancer patients can perceive the surgery as a threat more than the younger ones, since elderly patients are more at risk for intra- and post-operative complications and recover after the surgery with more difficulties. Detecting depressive symptoms in this oncological population appears important, since they are typically associated with a significant decrease in the quality of life, a deterioration in physical activity, difficulties in the relationships, and more intense pain [25]. Finally, the data evidenced that a lower educational level was associated with a greater presence of anxious symptoms, and this result is in line with the literature [26]. Thus, in the initial phase after the diagnosis, this factor may have a role with respect to the psychological distress, interfering with the searching for appropriate information on the medical aspects of the disease and of the care processes and services. However, the educational level can be associated with socio-economic conditions that can influence the levels of psychological distress related to the illness. Moreover, an inter-generational reflection would let us hypothesize that people with a middle school diploma are generally older than those with a high school diploma or a degree. Therefore, this association could be explained by the fact that most of the participants with a middle school diploma were elder people and widows, with the abovementioned implications. As underlined by Taskila et al. [27], the occupational state can play an ambivalent role in the development of an anxious and/or depressive symptomatology, configuring itself as a risk factor or as a protection factor depending on the interaction of various factors, such as, for example, seniority of work, type of job, the level of cancer-related fatigue, and possible cognitive impairment in the psychological functioning of the patient [28]. Furthermore, albeit in a different context, the scientific literature underlines how the presence of sons can become both a source of support and motivation for the treatment, and also a source of concern for patients with breast cancer [29]. In consideration of this and based on the results obtained from this study, the occupational status and the presence of sons cannot be considered a priori risk or a protective factor, but they must be always considered in a multifactorial perspective and contextualized in the subjective living condition of the patient. The limitation of this study is that, since the participants were all newly diagnosed women with breast cancer, the results cannot be generalized to other types of cancer and phases of the disease. Further studies should consider other cancer populations and investigate other socio-demographic factors (such as nationality, ethnicity, and the socio-economic level of the subjects) as long as the impact of the different disease’s stages on the development of any forms of psychopathology are considered, as well.

## 5. Conclusions

Older women, widows, and those with a low educational level seem to be more affected by more psychopathological distress during the phase between the diagnosis and the treatment. These data suggest considering these socio-demographic characteristics as factors of greater vulnerability in women newly diagnosed with breast cancer. Considering the impact of the psychological distress on the health and outcomes of the cancer care, it is important to identify the patients that, due to specific socio-demographic characteristics, are more psychologically vulnerable.

## Figures and Tables

**Table 1:** Socio-demographic characteristics of the sample (*N* = 478).

Socio-Demogaphic Characteristics	*n* (%)	
	Mean ± SD	
Age	58.19 ± 12.65	
Sons	Yes	352 (73.6)
	No	126 (26.4)
Marital status	Married/cohabiting partner	332 (69.5)
	Single	64 (13.4)
	Divorced	14 (2.9)
	Widow	68 (14.2)
Education	Primary school	77 (16.1)
	Middle school	167 (34.9)
	High school	182 (38.1)
	Degree	52 (10.9)
Occupational status	Employed	218 (45.6)
	Unemployed	21 (4.3)
	Housewife	96 (20.1)
	Retired	143 (30.0)
Nationality	Italian	466 (97.5)
	Europe	2 (0.4)
	Extra-Europe	10 (2.1)

Note: *N*: Absolute frequencies; %: Percentage; SD: Standard deviation.

**Table 2:** Contingency table with frequencies of patients above/under the HADS-A cut-off scores and the marital status.

HADS-A Groups	Frequencies, %, Adj res	Married/Cohabiting Partner	Single	Divorced	Widow	Total
		(*N* = 332)	(*N* = 64)	(*N* = 14)	(*N* = 68)	(*N* = 478)
**HADS-A** **Under cut-off**	*n*	205	44	11	29	289
	% in HADS-A	71%	15.22%	3.8%	10%	100%
	% in marital status	61.77%	68.75%	78.57%	42.64%	60.5%
	% of the total	42.9%	9.2%	2.3%	6.1%	60.5%
	Adj res	+0.9	+1.5	+1.4	−3.2	
**HADS-A** **Above cut-off**	*n*	127	20	3	39	189
	% in HADS-A	67.2%	10.58%	1.58%	20.63%	100%
	% in marital status	38.25%	31.35%	21.42%	57.35%	39.5%
	% of the total	26.6%	4.2%	0.6%	8.1%	39.5%
	Adj res	−0.9	−1.5	−1.4	+3.2	
**Total**	*n*	332	64	14	68	478
	% in HADS-A	69.45%	13.35%	2.92%	14.23%	100%
	% in marital status	100%	100%	100%	100%	100%
	% of the total	69.45%	13.35%	2.92%	14.23%	100%

Note: *N*: Absolute frequencies; %: Percentage; % in HADS-A: Row percentages; % in marital status: Column percentages; Adj res: Adjusted residues.

**Table 3:** Contingency table with frequencies of patients above/under the HADS-D cut-off scores and the marital status.

HADS-D Groups	Frequencies, %, Adj res	Married/Cohabitant	Single	Divorced	Widow	Total
		(*N* = 332)	(*N* = 64)	(*N* = 14)	(*N* = 68)	(*N* = 478)
**HADS-D** **Under cut-off**	Frequency	253	50	8	42	353
	% in HADS-D	71.67%	14.16%	2.26%	11.9%	100%
	% in marital status	76.2%	78.12%	57.14%	61.76%	73.9%
	% of the total	52.9%	10.5%	8.8%	1.7%	73.9%
	Adj res	+1.8	+0.8	−1.4	−2.4	
**HADS-D** **Above cut-off**	Frequency	79	14	6	26	125
	% in HADS-D	63.2%	11.2%	4.8%	20.8%	100%
	% in marital status	23.79%	21.87%	42.85%	38.23%	26.12%
	% of the total	16.52%	2.9%	5.4%	13.3%	26.12%
	Adj res	−1.8	−0.8	+1.4	+2.4	
**Total**	Frequency	332	64	14	68	478
	% in HADS-D	69.4%	13.4%	2.9%	13.3%	100%
	% in marital status	100%	100%	100%	100%	100%
	% of the total	69.4%	13.4%	2.9%	13.3%	100%

Note: *N*: Absolute frequencies; %: Percentage; % in HADS-D: Row percentages; % in marital status: Column percentages; Adj res: Adjusted residues.

**Table 4:** Contingency table with frequencies of patients above/under the HADS-A cut-off scores and the level of education.

HADS-A Groups	Frequencies, %, Adj res	Primary School	Middle School	High School	Graduate	Total
		(*N* = 77)	(*N* = 167)	(*N* = 182)	(*N* = 52)	(*N* = 478)
**HADS-A** **Under cut-off**	Frequency	43	88	119	39	289
	% in HADS-A	14.87%	30.44%	41.2%	13.49	100%
	% in education	55.8%	52.69%	65.38%	75.0%	60.5%
	% of the total	9.0%	18.4%	24.9%	8.2%	60.5%
	Adj res	−0.9	−2.6	+1.8	+2.2	
**HADS-A** **Above cut-off**	Frequency	34	79	63	13	189
	% in HADS-A	18%	41.79%	33.34%	6.87%	100%
	% in education	44.15%	47.3%	34.6%	25.0%	39.5%
	% of the total	7.15%	16.5%	13.2%	2.7%	39.5%
	Adj res	+0.9	+2.6	−1.8	−2.2	
**Total**	Frequency	77	167	182	52	478
	% in HADS-A	16.1%	34.93%	38.07%	10.9%	100%
	% in education	100%	100%	100%	100%	100%
	% of the total	16.1%	34.93%	38.07%	10.9%	100%

Note: *N*: Absolute frequencies; %: Percentage; % in HADS-A: Row percentages; % in education: Column percentages; Adj res: Adjusted residues.

## Data Availability

The data presented in this study are available on request from the corresponding author.
